# Respectable Challenges to Respectable Theory: Cognitive Dissonance Theory Requires Conceptualization Clarification and Operational Tools

**DOI:** 10.3389/fpsyg.2019.01189

**Published:** 2019-05-29

**Authors:** David C. Vaidis, Alexandre Bran

**Affiliations:** Laboratoire de Psychologie Sociale, Institut de Psychologie, Université Paris Descartes—Sorbonne Paris Cité, Paris, France

**Keywords:** cognitive dissonance, replication crisis, operationalization, measurement, theory, methodology

## Abstract

Despite its long tradition in social psychology, we consider that Cognitive Dissonance Theory presents serious flaws concerning its methodology which question the relevance of the theory, limit breakthroughs, and hinder the evaluation of its core hypotheses. In our opinion, these issues are mainly due to operational and methodological weaknesses that have not been sufficiently addressed since the beginnings of the theory. We start by reviewing the ambiguities concerning the definition and conceptualization of the term *cognitive dissonance*. We then review the ways it has been operationalized and we present the shortcomings of the actual paradigms. To acquire a better understanding of the theory, we advocate a stronger focus on the nature and consequences of the cognitive dissonance state itself. Next, we emphasize the actual lack of standardization, both in the ways to induce cognitive dissonance and to assess it, which impairs the comparability of the results. Last, in addition to reviewing these limits, we suggest new ways to improve the methodology and we conclude on the importance for the field of psychology to take advantage of these important challenges to go forwards.

## Introduction

Among the major theories in psychology, Cognitive Dissonance Theory (CDT; Festinger, 1957) holds a honorable position (Haggbloom et al., 2002; Devine and Brodish, 2003; Gawronski and Strack, 2012; Kruglanski et al., 2018). For more than six decades, CDT suggests that cognitive inconsistency leads to a motivational state that promotes regulation, which comes mainly through a change of opinions or behaviors. Many investigations of this theory have relied on the inconsistency between attitudes and behaviors, usually resulting in an attitude shift toward more consistency with the behaviors (e.g., Festinger and Carlsmith, 1959). Despite the quantity of publications supporting the model (Harmon-Jones and Mills, 1999; Vaidis, 2014; Harmon-Jones, 2019), and despite our deep attachment to this theory, we consider that research on CDT presents flaws which call into question the relevance of the methodology underpinning the theory. In the present paper, we stress and list what appear to us as major issues threatening the validity of CDT and we suggest means to cope with them. Finally, we invite the field to take advantage of these important challenges to go forward, and thus improve or complete the whole theory.

## A General Need for Clarification

About twenty-six centuries before our time, Sun Tzu's philosophy stated that “If you know the enemy and know yourself, you need not fear the result of a hundred battles.” Knowledge of weaknesses is just as important as awareness of strengths. Similarly, scientists should be alert to methodological flaws surrounding their models so as to confront empirical challenges serenely.

Recently, several important theories which contributed to social psychological knowledge were partially discarded or relegated to a secondary role (Open Science Collaboration, 2015). This has been the case for ego depletion theory (Hagger et al., 2016), as well as for priming effects on impression formation (McCarthy et al., 2018), and cognitive performance (O'Donnell et al., 2018). These revisions follow from the methodological and replication crisis started in 2011 (see Nelson et al., 2018), a landmark year for social psychology with Bem's publication (Bem, 2011) that raised a statistical and methodological shield wall, and with the exposition of a renowned scholar's fraud (Levelt Noort and Drenth Committees, 2012) that brought to light the existence of dishonest practices. The field reacted by increasing standards for scientific evidences in social psychology. For some, this process has been perceived as questionable (e.g., Schwarz and Clore, 2016) and sometimes as harsh (Fiske, 2016), and has sometimes been called the “data police,” the “inquisition” or even “methodological terrorists.” However, one reacts to the affliction, we consider that these hard times contribute to the debate and improve psychological science. Indeed, questions about the methodology of a field and requests for meta-analyses or multiple laboratory replications should not be seen as frightening enemies but as valuable assets, with the only purpose to contribute to a clarification of what is real, reliable, and could constitute solid knowledge for the future of social psychology.

In this context, does CDT needs to worry about its future and could it be reclassified from major league to classic-but-wrong-theory? Until now, replication projects have not yet focused on CDT and have spared this theory. But there is no reason to rejoice. We did definitely learn a lot from the six decades of existence of CDT and it influenced many fields and theoretical descendants (e.g., Aronson, 1992; Harmon-Jones and Mills, 1999; Gawronski and Strack, 2012). However, even the most fervent proponents of the theory –including ourselves– should admit that the field has avoided addressing some major criticisms which persisted through the years and that are still relevant today. These questions echo insistently in this time of methodological crisis, and we believe that the field should make a special effort to address them. Moreover, as one of the rare social psychology theories that propose a general pattern characterizing the human psyche and construction of reality, CDT is a very important theory for the field. This status should motivate scrupulous research and evaluation. Despite its status as the old lady of the discipline, CDT should be questioned as thoroughly as a young theory.

In the following sections, we discuss the weakness of CDT operationalization and suggest methodological improvements. In our opinion, these major issues have to be addressed, and focusing on these points should help the theory, the field and the whole discipline, to move forward.

## The Operationalization Issue: Problems and Ways Forward

Festinger (1957) states that non-fitting relations among cognitions generate a state of discomfort, now generally considered as involving negative arousal, that motivates people to cope with this situation, typically by adjusting one cognition to the other. The term he used to refer to this state of discomfort was dissonance. To stress the homeostatic nature of dissonance, he made a parallel with hunger: Deprived of food, people feel hungry and find a way to cope with their hunger. However, as if the same construct defined food deprivation and hunger, Festinger used the term dissonance for both the triggering relation and the state of discomfort that occur. Although CDT has been extensively revised, the original theory is still a central point of agreement and constitutes the core of the theory^1^ (see Vaidis, 2014; Harmon-Jones, 2019). Two main issues have ensued from this overlap: one regarding the definition of dissonance and one regarding its operationalization. Some additional issues to address follow from this: the key variables suffer serious theoretical misconceptions and the lack of methodological standardization restrains breakthroughs.

### Definition of “Dissonance”

#### One Term for One Concept: Dissonance or Inconsistency?

In science, it is normative and considered appropriate to use specific words to define specific concepts. A primary issue with CDT concerns the ambiguous term *dissonance*. In his original publication, Festinger (1957) used the term *dissonance* to refer to three different entities: the theory itself, the triggering situation and the generated state. This single terminology is still commonly used today and leads to imprecisions in studies (e.g., Martinie et al., 2017; McGrath, 2017; Cancino-Montecinos et al., 2018). Common sense suggests to consider using three different terms to define these entities. To improve clarity, Vaidis and Bran (2018) suggested calling the trigger *inconsistency*, the evoked arousal a *cognitive dissonance state* (CDS) and the theory *cognitive dissonance theory* (CDT).

The use of the term *inconsistency* to point out the presence of unfitting relations has already been proposed in the literature (e.g., Harmon-Jones, 2002; Gawronski and Strack, 2012). However, the state of cognitive dissonance, or CDS, is not always distinguished from the term for the theory, and they should be clearly differentiated. Various more or less precise suggested alternatives exist. Proulx and Inzlicht (2012), for instance, mischievously suggest *disanxiousuncertlibrium* as a term for the state of cognitive dissonance, while Harmon-Jones et al. (2009) suggest keeping the term *dissonance* for the state and refer to *cognitive discrepancy* for the triggering situation. Although the use of a unique terminology would definitely improve clarity, our point here is not to specify the consensual terms to be used, but rather to emphasize the necessity of using specific terms to designate distinct concepts instead of relying on one general term such as dissonance.

Refining the terminology used in CDT could not only clarify the theory, but also impact the whole conception of the theory regarding ways to cope with “dissonance” (Vaidis and Bran, 2018). In Festinger's view (1957), regulation strategies are supposed to reduce “dissonance,” but does that mean resolving the inconsistency or does that mean alleviating the arousal? This issue is never clarified in the original presentation of the theory, and differences in scholars' implicit definitions could result in radically different views about the nature of dissonance regulation. In our view, avoiding a confusing conceptualization of CDT requires specifying that the regulation strategy aims at CDS and not necessarily directly at the inconsistency. To serve that purpose, the term *regulation* fits best with the idea of generally decreasing the motivational state, while the term *reduction* could be reserved for regulation specifically aimed at reducing the inconsistency. In our opinion, this terminology is more integrated with the general theory (see Vaidis and Bran, 2018), as well as more connected to current knowledge (see also Proulx et al., 2012; Jonas et al., 2014; Levy et al., 2017).

### Assessing Reduction Is not Assessing “Dissonance”

Investigating strategies for reduction has historically been the overwhelming focus of CDT research. For decades, studies have been focused only on attitude change (for historical reviews, see Vaidis and Gosling, 2011; Vaidis and Bran, 2018), but the regulation strategies can be numerous (see McGrath, 2017). Traditionally, regulations are used to infer the existence of the CDS and authors reason that if individuals have changed their attitude, then they must have experienced cognitive dissonance (Devine et al., 1999). A fundamental perspective we take in this paper is that attitude change is only a means of regulation that occurs in specific conditions, but is not a synonym for CDS, nor is any other regulation strategy. Assuming an equivalence between the occurrence of regulation and the existence of a CDS is a logical error and must be avoided. Indeed, if the process conceptualized by CDT involves three steps (inconsistency-CDS-regulation), then regulation is only the third part of a triptych causal-relation. Because there is a constellation of possible regulation strategies and many variables are supposed to influence them (McGrath, 2017; Vaidis and Bran, 2018), the absence or presence of any given mode of regulation neither confirms nor disconfirms the presence of a CDS^2^.

While attitude and behavior change are the regulation strategies that have been the most studied, plethora of regulations can occur, including, for instance, trivialization (Simon et al., 1995), denial of responsibility (Gosling et al., 2006), self-affirmation (Steele and Liu, 1983) or even value affirmations (Randles et al., 2015). Given the number of possible regulation strategies, assessing only one of them limits the conclusion that can be drawn. For instance, the absence of use of a single strategy does not suggest that no regulation has occurred through others, even more as we know very little about what influence the choice of a strategy (Weick, 1965; McGrath, 2017; Vaidis and Bran, 2018). Hence, a serious assessment of regulation strategies that avoid false negatives would have to include all possibilities. Because it is difficult to predict which strategy will be used, it seems unreliable to postulate the existence of CDS and its magnitude on the sole basis of the use of a regulation strategy.

Collectively, the methodological issues concerning assessment in CDT invite consideration of the examination of regulation as a secondary goal for now. As a first step, it seems essential to direct efforts at the development of a clear instrument for measuring the CDS before expecting a clear relation with regulation. These points will be developed further.

### The Right Operationalizations to Test the Theory

A particular strength of CDT is the simplicity of its main hypotheses. The detection of an inconsistency arouses a state of discomfort (i.e., CDS) that motivate the individual to reduce it. So how to test such theory? Experimental method suggests to manipulate the hypothesized responsible variable and to assess the suspected effects. This seems trivial from a methodological point of view, but most paradigms in cognitive dissonance do not comply with this principle. Indeed, classic paradigms in CDT manipulated, for instance, the pay for a discrepant behavior (Festinger and Carlsmith, 1959), the severity of the pressure to inhibit a behavior (Aronson and Carlsmith, 1962), or the deployed effort to join a group (Aronson and Mills, 1959). From a theoretical and methodological point of view, these variables are not manipulations of inconsistency but moderator variables linked to the situation (i.e., incentive, justification, effort) that decrease or increase the CDS (Festinger and Carlsmith, 1959, pp. 203–204). Indeed, cognitions consistent with the behavior (presumed the most resistant) are supposed to decrease the magnitude of the CDS, while inconsistent ones are supposed to increase it. These variables are thus supposed to modulate the magnitude of the CDS and, in some specific cases (i.e., commitment), they bolster the resistance to change and thus orientate the occurrence of reduction strategies (Vaidis and Gosling, 2011; Vaidis and Bran, 2018). Therefore, these moderators can influence the magnitude of dissonance but do not constitute a manipulation of the inconsistency, as would be comparing an inconsistent situation to a neutral or consistent one.

In this vein, the commitment variable is the archetype of a confounded variable. When using a counter-attitudinal task, the central variable since Linder et al. (1967) turns out to be commitment, mainly manipulated through choice (e.g., Zanna and Cooper, 1974; Elliot and Devine, 1994; Simon et al., 1995). Within this framework, problematic behaviors that are freely chosen (i.e., High choice) are the “dissonance condition” while the same behaviors occurring under pressure (i.e., Low choice) are supposed to be the “no-dissonance condition.” Therein lies the rub: the choice variable is fundamentally distinct from inconsistency (see Kiesler, 1971). In fact, these experiments do not study how people react to an inconsistency, they study how commitment (via choice) influence people's reaction to an inconsistency. In other words, we want to stress that inconsistency without commitment is still inconsistency, and that the commitment variable is first and foremost a factor that will influence the resolution (Kiesler, 1971; Vaidis and Gosling, 2011). Early warnings of this error were made in the past (Chapanis and Chapanis, 1964; Kiesler, 1971; Festinger, 1987/1999), but this issue is still present today, as commitment continues to be the paradigmatic variable in many recent publications (e.g., Blackman et al., 2016; Martinie et al., 2017).

This issue could be fixed by redefinition of core concepts and a paradigm change for a systematic manipulation of inconsistency. The easiest way to achieve this would be to compare counter-attitudinal tasks to neutral or pro-attitudinal tasks. Some recent paradigms have indeed shifted their focus to the manipulation of inconsistency. For instance, the hypocrisy paradigm (Aronson, 1992; Stone and Fernandez, 2008; Priolo et al., 2019) compares inconsistent conditions to neutral or consistent ones. Likewise, some new paradigms focus on minimal inconsistencies, that is, inconsistencies that involve very few cognitions other than the inconsistency per se (e.g., Levy et al., 2017), and compare inconsistent conditions to neutral ones. These new paradigms are encouraging, but researchers in the field must still clearly realize that varying factors such as commitment is not the same as varying inconsistency.

### Inconsistency: Operationalization of Both Manipulation and Measure

Variable operationalization refers to two distinct things (e.g., Leary, 2014): on the one hand, it could be the translation of a variable into experimental language, and on the other hand, it could refer to the measurement of said variable. For instance, it can be the means to manipulate hunger in an experimental setting and also the measurement of such hunger. Both operationalizations provide important means to assess a model and, concerning CDT, both need refinement.

Given that CDT deals with inconsistency, one should systematically ensure that there is inconsistency, and ideally assess it. Indeed, the model suggests a relation between the variables involved in the inconsistency, the CDS and its regulation. Festinger considered that the “magnitude of the dissonance will be a function of the importance of the elements” (p.16, 1957/1985). Hence, a higher level of CDS is evoked when the involved cognitions are important (Festinger, 1957). For instance, exposure to slight belief disconfirmation would generate a lower degree of CDS than exposure to a strong disconfirmation. Similarly, being forced to kill a snail should raise a somewhat lower CDS than having to kill a cute kitty. As an indicator, we bet that the last part of the previous sentence has generated a more intense reaction to the readers. This is because the involved elements are subjectively more important, and thus generate more CDS.

The relation between inconsistency and CDS is more than a presence-absence relation and it forms a main axiom of CDT. As a consequence, to achieve a test of the model and clear predictions, one must measure the degree of inconsistency or other factors responsible for its magnitude which are supposed to impact the CDS (e.g., strength, importance, centrality). This relation between inconsistency and CDS has been under-examined in the literature, and an effort must be made to operationalize inconsistency rigorously. This means that operationalization of both the assessment of inconsistency and the manipulation of inconsistency is required, and that only systematic measures would allow for investigation of the relations between inconsistency, CDS and the regulation process. Moreover, in the present state of conceptualization, assessing the inconsistency may also be the most relevant way to assess the “dissonance” construct. As a consequence, resolving the issue of the relation between inconsistency and the CDS could be achieved by using conditions that involve several degrees of inconsistency (e.g., low; medium; high), assessing it, and by measuring the CDS generated by these different conditions.

### On the Nature of the Cognitive Dissonance State

In a seminal paper, Elliot and Devine (1994) made a major advance by confirming the existence of discomfort prior to attitude change (Exp. 1) and a decrease of such discomfort following the attitude change (Exp. 2). This paper stressed a fundamental point by examining the hypothesized state, but several questions remain concerning the nature and the exact role of the CDS. Indeed, at our knowledge, the existing studies examining the CDS are subject to the same methodological issues we raised previously, and the field lacks a reliable instrument to assess the CDS. Further research is crucial to define and explore the exact nature of the CDS.

Concerning the nature of CDS, we still know quite little. By nature, we mean the parameters that allow a clear definition of this “state,” such as the experience of a specific emotion or the state's intensity, valence or motivational capacity. Conceptually, Festinger (1957) defined cognitive dissonance as a state of psychological discomfort that motivates its regulation, then later, as a state of arousal (e.g., Lawrence and Festinger, 1962). Likewise, other authors have described the CDS as a state of tension (Croyle and Cooper, 1983; Kruglanski and Shteynberg, 2012), an unpleasant feeling (Harmon-Jones, 2000), or a state of aversive arousal (Proulx et al., 2012). From all these definitions, it is not clear if the CDS is supposed to be a distinct and specific state, or if it can be expressed by emotions. Several researchers have for instance considered *guilt* (Stice, 1992), *surprise* (Noordewier and Breugelmans, 2013), and *anger* (Geschwender, 1967) as evidence of CDS. However, this view is not consensual. When constructing a self-report questionnaire, Elliot and Devine (1994) used only three items to assess the nature of CDS (*uncomfortable, bothered, uneasy*), excluding many other items, such as *guilt*. They emphasized afterwards that different affect assessments could capture the nature of cognitive dissonance depending on the situation (Devine et al., 1999). Despite this clarification, most researchers using the scale continue to use the index in its original form, thus dissociating CDS from the other items (Galinsky et al., 2000; Harmon-Jones, 2000; Norton et al., 2003; Monin et al., 2004; Vaidis and Gosling, 2011). In another perspective, Kenworthy et al. (2011) have suggested that guilt could be the most relevant predictor of dissonance effects instead of a specific CDS, thus making a clear distinction between the two. For their part, Gosling et al. (2006) used the Elliot and Devine's scale but relied on negative-self oriented affect to assess dissonance instead of their dissonance specific index. While these different views co-exist in the literature, there has not been a clear debate yet on the nature and the specificity of the CDS. Altogether, according to the diversity of specific emotion studied, focusing on one specific affect or on a cocktail of affects to capture the nature of CDS seems inappropriate.

Another perspective is to consider CDS as a non-specific emotion and to look for more general features of CDS. In this perspective, most agree on a negative valence and an aversive feature (i.e., individual are motivated to avoid it). Nevertheless, it is not so obvious and could even be debated. Actually, the field has rarely set up situations that could evoke something other than a negative valence: Most studies deal with undesired inconsistencies (i.e., writing against what you want; being exposed to undesired information), while there is a lack of data about affect evoked by an inconsistent but positive cognition (e.g., performing better than expected; Aronson and Carlsmith, 1963). In a recent model, Kruglanski et al. (2018) suggest that the evoked affect could differ depending on expectancy and desirability of outcomes. This assumption implies that disconfirmation of a positive expectancy generates a negative affect while the disconfirmation of a negative expectancy generates a positive affect. An interesting parallel could be drawn with *surprise* (see Noordewier et al., 2016): the initial detection of surprise has a negative valence, but the final valence depends on the valence of the outcome. In a similar vein, Martinie et al. (2013) demonstrated a temporality of valence: assessing facial activity, the initial reaction to dissonance is undifferentiated and it is only after some time that negative valence appears. This invites the examination of the nature of the CDS by taking into account a time course.

Finally, another possible feature of CDS concerns its relation to action tendencies. For the Action-Based Model (ABM; Harmon-Jones, 1999; Harmon-Jones et al., 2015), CDT serves the ultimate goal of reducing the interference with effective and unconflicted action. As a consequence, the CDS is supposed to be activated when it conflicts with action and triggers an approach-oriented state. This model is supported by several observations including neural activation of zones linked to conflict and its resolution, such as the anterior cingulate cortex (Harmon-Jones et al., 2008a,b; van Veen et al., 2009; Izuma and Murayama, 2019). However, some models suggest that approach is not necessarily the sole answer. Based on an extensive analysis of low level processes in reaction to threat, Jonas et al. (2014) assume that inconsistency triggers at the very beginning the inhibition system (BIS) and then, if no resolution has occurred, only in a second step is the behavioral approach system (BAS) activated. Hence, once more the time course could be relevant to understand the CDS process.

In regard to current knowledge, we have to admit that despite CDS being the core of the model, we know very little about it. Its affective properties are unclear and the time course is yet understudied. We believe that the intensive examination of its nature is necessary to develop an operational assessment of the CDS, which is a fundamental requirement before drawing further conclusions about the CDT. Finally, as noted previously, the diversity of the induction tasks could also explain a large part of the variance in the observed nature of CDS and the lack of clear paradigms limits current understanding. Additionally, a large part of the studies examining the nature of dissonance rely on a manipulation of choice instead of inconsistency, which could partially bias the conclusions. The specificity of elicited emotions, valence or action tendencies could depend on the task, but also on the operationalization and on the design, and one could hypothesize either that the specific state or regulation of it could stem from the specificity of the induction. So in addition to a better operationalization and a better measurement instrument, we also call for better standardization.

### Arguments for Standardization of Procedures

Paradigms can be defined as scientific “traditions”: models, techniques and expected results. While they can be good or bad for the evolution of science (see Kuhn, 1962), their main interest is that they are supposed to reduce the variations to the minimum level to permit evaluation of the outcomes in a cumulative science perspective. Concerning CDT, for years, the general paradigm was based on the manipulation of choice and the assessment of the attitude, although in the same time many other points were subject to variation. For instance, the importance of the involved cognitions (e.g., the topic) varied greatly from a study to another, as well as the presence and kind of control condition (e.g., without inconsistency, consistency), or the assessment of the CDS. We consider that a paradigm shift taking more into account the central variables of the theory would be an important step. In addition, the standardization of both the induction and the assessment would help in testing the core hypotheses of the theory.

### Standardization of the Induction Task

The CDT field is fruitful, with hundreds of studies covering a large array of tasks and topics. This number of studies is a strong argument for the conceptual validity of the theory. However, some of the core hypotheses of CDT have not been as thoroughly examined and, in their case, the field may benefit from an increased standardization. One of our main concerns here is about the CDS and its investigation. Overall, cognitive dissonance studies have many variations with one another. For instance, counter-attitudinal essays have been investigated with various topics and many differences concerning the instructions, the time course (e.g., length, temporal distance between the induction and the assessments) and the task (e.g., argument, essay, speech). In addition, these studies are strongly socially contextualized and thus may have different impacts depending on place, culture, and temporality. All these variations are likely to alter a number of variables theoretically linked with the CDS and its regulation, such as the importance of the involved cognition, the evoked emotions, the level of self-involvement, or the perceived choice. As we emphasized above, this large variation in the induction is beneficial for the conceptual validity of the theory. However, all these variations can also be impairments when trying to study some specific hypotheses, such as those about the nature and role of the CDS, and its regulations. Each variation between two studies creates room for a potential confounded variable.

In the same vein, the nature of the induction could be fundamentally different from one another (e.g., counter-attitudinal essay, hypocrisy paradigm, free-choice paradigm). These differences can have important impacts on the following assessments. For instance, we developed previously that the CDS may be linked to various emotions, such as *guilt* or *surprise*. Depending on the nature of the induction, it seems logical that some emotions may be more evoked than others. For instance, when someone deliberately and publicly accepts to write a counter-attitudinal essay, or remembers behaviors that are inconsistent with previously preached values, this is more likely to evoke *guilt*. In the meantime, seeing a perceptual anomaly is more likely to be associated with *surprise*. An interesting perspective is also to consider inconsistencies that could evoke a positive valenced emotion, such as expecting a low grad on an exam and receiving a high one (Gawronsky and Branon, 2019). If there is a common CDS to all these situations then its investigation is made particularly difficult in the actual variation in the inductions, and it also makes more complex the examination of the regulation process as these emotions could promote different strategies (Higgins, 1987; Devine et al., 1999; Niedenthal et al., 2006). The same reasoning applies when assessing the CDS through physiological measures. Because several emotions are likely to be evoked, how to distinguish the physiological activity associated to the CDS from the “noise” due to these emotions? Actually, there seems to be some variabilities here depending on the nature of the induction: counter-attitudinal essays have been related to increased GSR (Croyle and Cooper, 1983; Elkin and Leippe, 1986), but feedbacks inconsistent with expectancies have not (Etgen and Rosen, 1993). Likewise, the free-choice paradigm has been related to elevated heart-rate (Etgen and Rosen, 1993), but counter-attitudinal essays have not (Croyle and Cooper, 1983).

We think therefore that the field would benefit from increased standardization. This standardization of the induction would rely on both a better operational definition of the manipulated variables, as we mentioned above, and also on the report of variables that are likely to influence the CDS, and thus its regulation. For instance, the hypocrisy paradigm has been investigated with very different topics and methods, and Priolo et al. (2019) found no evidence in their meta-analysis for the existence of a CDS. This conclusion could question either the theory or either the relevance of the methodology. These authors stress the lack of available studies and their important variations to explain this null result, what is in line with our concerns.

In our opinion, we consider that more standardization could permit to examine such specific hypotheses and to investigate more precisely the effects. To achieve this goal, and reduce variation between studies, the standardization of the induction would also require moving away from tasks grounded in social background, temporal, or cultural references. This would reduce many biases and allow multilab studies. Also, to capture fine variations with lower noise and to be able to modelize the process, these requirements suggest movement toward lower level processes. It could require to look at the very minimal prerequisite for CDT, that is to manipulate inconsistency while the other socially contextualized variables are reduced to their strict minimal (e.g., commitment). Finally, in accordance with the operationalization issue, one of the first thing to assess is probably inconsistency, which could permit more relevant comparisons between studies and help to correct local or individual biases. The assessment of additional parameters that could influence the CDS and its regulation (e.g., self-involvement) may also facilitate the investigations and ultimately permit to estimate the independent effects due to each of these variables. Finally, in addition to this standardization of the induction task, one has to rely on standard assessment of the CDS.

### Standard Assessment of Cognitive Dissonance State

Reliable tools are necessary to examine the nature of the CDS. Because the CDS is the core motive of the model and could vary depending on the induction situation, we must get closer to standardized instruments. The prevalence of a unique tool should permit comparison and reliable expected effects (i.e., size and quality).

Explicit self-reported scales have been useful at times (e.g., Elliot and Devine, 1994) but present limits. Indeed, they imply that individuals can consciously and accurately assess and report their emotions. Moreover, there is a lack of standardization in the field in the instruments that are used. For instance, even when referring to the same scale, scholars use different methods of scoring, different instructions, and even different sets of items. This absence of standard rules favors *HARKing* (Kerr, 1998) in the choice of indicators for CDS.

We identify three main perspectives on capturing the CDS. One classic approach relies on assessing the specific emotion. By doing so, it suggests listing the affects that could fit with cognitive dissonance and assessing all of them. This is probably the worst perspective because the affect may vary depending on the nature of the task and because this method mostly relies on individuals to reliably assess their emotion, which is not ensured (Niedenthal et al., 2006). A second option is to assess the specific features of the CDS. It implies sharply defining these features (e.g., valence, aversivity, intensity, action tendency). It also requires taking into account the time course, as it is supposed to be relevant. Finally, a last and complementary perspective relies on physiological proxies. Not so distinct from measurement of subjective intensity, its main interest is that this can be done in parallel to the previous approaches to provide convergent validity. Finally, the development of an efficient tool to assess the CDS should probably rely on multiple measures.

Three other points seem necessary to achieve this standardization: Assessment of the CDS must be (a) non explicit, (b) non-invasive, and (c) follow open science principles. (a) By non-explicit, we mean that the respondent should be unaware of what is being assessed. Otherwise, participants may falsely reports their feelings, for instance due to social desirability or attribution errors (e.g., Rosenthal and Rosnow, 1969; Nisbett and Wilson, 1977). For this purpose, the use of implicit assessments that are less likely to be influenced by awareness and conscious control, such as reaction time or implicit association (e.g., Nosek and Banaji, 2001; Quirin et al., 2009) could reliably assess the evocation of CDS (e.g., Levy et al., 2017). (b) By non-invasive, we mean that respondents should not be impacted by the assessment itself. Indeed, studies have shown that participants could misattribute the CDS to other sources (e.g., Zanna and Cooper, 1974). If participants attribute arousal to the measurement instrument, this could alter the regulation process (e.g., Croyle and Cooper, 1983). This point is specifically important for physiological measures which are useful for the achievement of a standardization tool. Considering the physiological measures, many have been invested in the past, such as GSR/EDA (e.g., Croyle and Cooper, 1983), heart rate (e.g., Gerard, 1967) or even *f* MRI (e.g., de Vries et al., 2014), but most are likely to be perceived as invasive and could trigger a misattribution process. An interesting development could stem from pupillometry, because this method does not require invasive apparatus nor a potentially threatening context (as would *f* MRI), and participants could be unaware of the assessment. Some initial results indicate that pupil dilatation is a potential proxy for the detection of inconsistency and could be used to capture the evocation of CDS as well as specific action mindset (e.g., Sleegers et al., 2015; Proulx et al., 2017). Although these two suggestions cannot efficiently capture the nature of the CDS, they could therefore present interesting methods to detect and assess the magnitude of CDS with a low odd of response bias and of misattribution.

Finally, according to (c) open science (e.g., Klein et al., 2018), the tool and more specifically the data have to be shared, accessible, and transparent. A major point is that raw data must be provided and publicly available. In our opinion, this is especially important for the current issues of CDT. For instance, investigating the time course of CDS and some of its influence could already be possible if authors shared their data along with the time course of their protocol. The affective nature of the CDS is another area that could be enlighten if more data were available and ready to be aggregated. It would help the field if data were collected with fully informed standardized designs and were accessible, thus allowing researchers to reach a sufficient amount of observations to fully understand the process.

## Testing the General Model

The general model of CDT suggests that the detection of an inconsistency will evoke a CDS, which will motivate a regulation strategy. Most investigations have focused on these regulation strategies, however they may have been done too early and some conclusions may have been drawn without sufficient understandings of the preceding parts of the model (see Weick, 1965; Greenwald and Ronis, 1978; Vaidis and Gosling, 2011). In the previous paragraphs, we made several suggestions for testing CDT in a more reliable way and, as the model is sequential, the suggestions should also respect a step sequence. Thus, regulation should be the last part of the examination, not the first. In addition, a serious evaluation of the theory requires assessing the whole model and not only the last sequential part. In the current state, the general model of cognitive dissonance (inconsistency-CDS-regulation) has to be put to the test. This consideration could imply reexamining many former conclusions drawn in the first decades of CDT. All the information gathered from this examination could provide rich understanding for the theory and help in reconnecting the CDT to the whole field.

### Verdict of Not Proven

Science requires time. Once the inconsistency induction and the CDS issues are fixed—and only after that—the research could finally focus seriously on the regulation sequence and the whole model. Indeed, the genuine model considers regulation to be driven by the CDS and thus the theory expects individuals to be motivated for regulation. Hence, with a clear operationalization of inconsistency and CDS, the total absence of regulation should be a refutation of the theory. But because assessing all the strategies is nearly impossible, it would be more interesting to examine the factors influencing the choice of regulation.

As expressed previously, while this assessment is premature for now, a comprehensive understanding of the former sequences could facilitate the identification of reliable factors that orientate the most likely strategies in a given situation. This evaluation could as well confirm some previously suggested factors (see McGrath, 2017) but also reassess a large part of the literature. However, this examination requires a step-by-step process, starting with the first sequence proposed and finishing with the regulation predictors. Finally, only the examination of the full inconsistency-CDS-regulation sequence will allow for testing the theory as a whole.

Until these prior steps are accomplished, studies cannot rely on regulation strategies as a unique cue for examining the model. More crucially, when the CDS is not present, the conclusions drawn from the only partial occurrence of one or two reduction strategies could bias assessment of the model. Of course, this does not preclude investigating specific strategies, such as attitude or behavioral changes, in a relevant applied setting. But we would like to stress that, given the current state of knowledge, studying regulation strategies has little interest for the evaluation of the general model or its improvement. Thus, this position does not imply avoiding measuring regulation, but stresses the necessity of assessing the CDS.

### With a Little Help From the Community

Examination of the theory's validity requires examining all the core fundamentals. Many hypotheses will require reexamination in light of new tools and knowledge, while some other core hypotheses are yet unexamined for now and could be crucial in the assessment of the model. To our knowledge, no studies have clearly investigated, for instance, the functional relation between the inconsistency-CDS-regulation triptych, this point being nevertheless a central element of the theory. Despite being central to the model, this expected positive relation function has not been seriously examined yet. Furthermore, examining the form of the relation is essential. However, testing such a model would require a huge amount of data with a high degree of precision, something that could only be attained with cooperation between cognitive dissonance scholars.

The examination of the theory will require high powered studies with strictly relevant variables. We do not recommend investing resources in large scale replication projects of earlier studies. Indeed, as we stressed previously, these studies have methodological flaws that limit their interpretation. Instead of replicating the errors made in the past, resources should be devoted to developing reliable, smart, and well-powered tests of the theory and of its hypotheses. To limit the bias, these designs should be computer based to avoid experimenter effects (e.g., Rosenthal and Rosnow, 1969). Using standardized scripts should also facilitate replications and variations all over the globe. Moreover, with a little help from the community, crowdsourced research tools (e.g., Collaborative Replications and Education Project, 2018; Moshontz et al., 2018) could allow a rapid, clear and high powered evaluation of the theory.

### Connectivity to Broader Theories

Finally, the suggested working plan is huge. What could this extensive examination provide for CDT and for the field of social psychology? The dissonance model is one of the few models to suggest a general base for human functioning, but regrettably there is a lack of connection with other fields. The overspecialization of its operationalization and its historically restricting paradigms could have explained part of this side-lining (e.g., Aronson, 1992; Swann, 1992). In a way, we assume that most will agree that CDT has deeply influenced the field and shaped the conception of major trends such as, for instance, attribution theories (Jones and Davis, 1965; Kelley, 1967), social cognition (Fiske and Taylor, 1984), motivated reasoning (Kunda, 1990), and self-regulation models (e.g., Scheier and Carver, 1988; Blascovich and Tomaka, 1996), but some would also contend that CDT is fossilized, with outdated paradigms, and distant from science, with tautological or unfalsifiable considerations (e.g., Lilienfeld, 2005; Griffin, 2012). With the recent methodological crisis, calls for conceptual cleaning and merging of general models have appeared. This could be the occasion for CDT to reconnect with broader theories and several attempts have already been made. For instance, the Meaning Maintenance Model (MMM; Heine et al., 2006; Proulx and Inzlicht, 2012) is a proposal for such merging. For the MMM, expectancy violation, mortality salience, or exposure to inconsistency all follow a common phenomenon of meaning violation that triggers the same neurocognitive and psychophysiological systems (see Jonas et al., 2014) and that motivates compensatory behaviors. This means that whatever the induction and the specific setting of the theory, the general process could be the same. Recent data (Randles et al., 2015) support that CDT is similar to many other meaning violations and such suggestions are real opportunities to gain a deeper comprehension of human functioning. Investigations of CDT in social neuroscience (e.g., van Veen et al., 2009; Harmon-Jones et al., 2015; Izuma and Murayama, 2019) also show similitudes in the activated areas with other theories. For instance, the *anterior cingulate cortex* is consistently activated in CDT paradigms, but also in MMM paradigms (see Proulx et al., 2012) and in mortality salience paradigms (Quirin et al., 2012). Last, suggestions about the induction procedure, such as the use of implicit inconsistency exposure (Levy et al., 2017), also permit merging several procedures which are widespread in close fields (e.g., Stroop task) but that were unusual for CDT.

The inclusion of CDT into broader models also proposes to expand our thoughts about the theory. From an evolution perspective, the process underpinning CDT should serve an important function to be present today. Moreover, it does not appear as a human specific process as many other species have shown evidence of cognitive dissonance (Egan et al., 2007, 2010; Harmon-Jones, 2017). For the action-based model of CDT (Harmon-Jones, 1999; Harmon-Jones and Harmon-Jones, 2002; Harmon-Jones et al., 2009), this process preserves the efficacy of action: when confronted with choices, regulating our attitudes toward these choices helps to go ahead and to promote action over inaction. In our personal view, one could also consider that the cognitive dissonance process serves the ultimate goal to control our environment: when exposed to events that confront expectations, a physiological reaction triggers the motivation for a regulation; that is to revise the expectations or reject the new information. Finally, cognitive dissonance process could have played an important role in the entire human evolution. Following this last perspective, Perlovsky (Masataka and Perlovsky, 2012; Perlovsky, 2013, 2017) considers that CDT could explain the fundamental role of music and prosody in humanity. In his view, music could be a means allowing to overcome CDS, thus it would have promoted the acceptance of new knowledge. Altogether, this promotes to reconsider CDT along with its connections to other psychological processes. This represents a wide area of research, but we think that there is much to gain in widening the scope of CDT.

## Conclusion

CDT is an old and respectable theory, but at the same time is still under construction. One can acknowledge the impressive contribution of this theory to psychology, but one cannot avoid recognizing that many critical questions remain and many methodological deficiencies are obviously present.

In the current paper, we exposed what we considered major flaws in the theory, which are mainly conceptual shortcomings and a need for stricter operationalization. Because a better understanding of the methodological flaws is important to future theoretical progress, we suggested some ways to address these shortcomings. Our essential take home message is, first, to focus on an operational distinction for the triptych elements of CDT, that is the inconsistency, the dissonance state (CDS) and the regulation strategies. In addition to investing effort in systematic and standard operationalization of these concepts, the examination of the whole model could deeply improve the theory and the understanding of human psychology.

Finally, looking on the bright side, social psychology is not suffering a decade of crisis. It is only leading the way for the next generation of researchers who will take steps to move the discipline toward stronger and more reliable knowledge about the human mind. As a final word, and as fervent supporters of CDT, we affirm that it is definitely an elegant theory. However, science should not concern itself with the gracefulness of a theory but only about the solidity of the evidence supporting it and its own falsifiability.

## Author Contributions

All authors listed have made a substantial, direct and intellectual contribution to the work, and approved it for publication.

### Conflict of Interest Statement

The authors declare that the research was conducted in the absence of any commercial or financial relationships that could be construed as a potential conflict of interest.

## Footnotes

^1^One could consider here that we are addressing an old point of view about CDT as the theory has been revised very early and very extensively since its beginning (for a review, see Harmon-Jones, 2019). However, all the revisions fit with Festinger's original statement, and while most of the revisions have been questioned (Greenwald and Ronis, 1978; Harmon-Jones et al., 1996; Vaidis and Gosling, 2011), the original statement has never been empirically proven wrong. For instance, while the required involvement of the self has been an important suggestion for the theory (e.g., Aronson, 1969, 1992; Steele and Liu, 1983), its role has then been considered as a potential moderator (Stone and Cooper, 2001), and even of secondary importance (e.g., Egan et al., 2007; Proulx et al., 2012). Some of the most recent views concerning CDT are even going back to Festinger's original statement (Gawronski and Strack, 2012; e.g., Levy et al., 2017). However, we should emphasize that this position is not yet consensual and most of the issues we raise are observable in the current publications of CDT.

^2^More formally, in a triptych logical relation A → B → C, if the universe of possible Ci is not finite, the observation of any Ci or non-Ci cannot state anything concerning B, and only the assessment of B permits confirming or disconfirming the causal relation.
